# Supplementary material for: Declining trend of smoking and smokeless tobacco in India: A decomposition analysis
Source: PLoS One. 2021 Feb 25;16(2):e0247226. doi: 10.1371/journal.pone.0247226 (PMC7906458; doi:10.1371/journal.pone.0247226)
Supplement: S1 File — (DOCX) [file pone.0247226.s001.docx]

**Supplementary File on Analysis**

The syntax for the Logistic Regression is as follows:

- **Logistic Regression for Smokers**

LOGISTIC REGRESSION VARIABLES Smoker_

/METHOD=ENTER Regions Residence_ NFAC1_2 Agegroups Gender_ Educationgroups Occupations

Illness_Smoker Stroke_smoker Heartattack_smoker Lungcancer_smoker Illness_smokeless

/CONTRAST (Regions)=Indicator(1)

/CONTRAST (Residence_)=Indicator(1)

/CONTRAST (NFAC1_2)=Indicator(1)

/CONTRAST (Agegroups)=Indicator(1)

/CONTRAST (Gender_)=Indicator(1)

/CONTRAST (Educationgroups)=Indicator(1)

/CONTRAST (Occupations)=Indicator(1)

/CONTRAST (Illness_Smoker)=Indicator(1)

/CONTRAST (Stroke_smoker)=Indicator(1)

/CONTRAST (Heartattack_smoker)=Indicator(1)

/CONTRAST (Lungcancer_smoker)=Indicator(1)

/CONTRAST (Illness_smokeless)=Indicator(1)

/PRINT=CI(95)

/CRITERIA=PIN(0.05) POUT(0.10) ITERATE(20) CUT(0.5).

- **Logistic Regression for Smokeless tobacco users**

LOGISTIC REGRESSION VARIABLES Smokeless_

/METHOD=ENTER Regions Residence_ NFAC1_2 Agegroups Gender_ Educationgroups Occupations

Illness_Smoker Stroke_smoker Heartattack_smoker Lungcancer_smoker Illness_smokeless

/CONTRAST (Regions)=Indicator(1)

/CONTRAST (Residence_)=Indicator(1)

/CONTRAST (NFAC1_2)=Indicator(1)

/CONTRAST (Agegroups)=Indicator(1)

/CONTRAST (Gender_)=Indicator(1)

/CONTRAST (Educationgroups)=Indicator(1)

/CONTRAST (Occupations)=Indicator(1)

/CONTRAST (Illness_Smoker)=Indicator(1)

/CONTRAST (Stroke_smoker)=Indicator(1)

/CONTRAST (Heartattack_smoker)=Indicator(1)

/CONTRAST (Lungcancer_smoker)=Indicator(1)

/CONTRAST (Illness_smokeless)=Indicator(1)

/PRINT=CI(95)

/CRITERIA=PIN(0.05) POUT(0.10) ITERATE(20) CUT(0.5).

**Decomposition**

**The following analysis has been carried out for each independent variable.**

The proportion was calculated using Univariate analysis for both GATS-1 (P1) and GATS-2 (P2).

The beta coefficient was taken from the Logistic Regression for GATS-1 (B1) and GATS-2 (B2).

Step 1: (P2 – P1) Proportion of GATS 2 subtracted from proportion of GATS-1

Step 2: (B2 – B1) Beta coefficient of GATS-2 subtracted form beta coefficent of GATS-1

Step 3: Calculation of Rate: Multiplication of proportion of GATS-1 into the difference obtained from Step 2. P1*(B2-B1)

Step 4: Calculation of Composition: Multiplication of Beta coefficient of GATS-1 into difference obtained from Step 1. B1*(P2 – P1)

Step 5: Calculation of Interaction: Multiplication of difference obtained in Step 1 and Step 2. (P2 – P1)* (B2 – B1)

Step 6: Calculation of proportion of overall change due to change in rates, composition and interaction.

Rate: Rate calculated in Step 3/ Sum total of Rate * 100

Composition: Composition calculated in Step 4/ Sum total of Composition * 100

Interaction: Interaction calculated in Step 5/ Sum total of Interaction * 100
