# Supplementary material for: Declining trend of smoking and smokeless tobacco in India: A decomposition analysis
Source: PLoS One. 2021 Feb 25;16(2):e0247226. doi: 10.1371/journal.pone.0247226 (PMC7906458; doi:10.1371/journal.pone.0247226)
Supplement: Attachment — Submitted filename: Reviewer response.docx [file pone.0247226.s003.docx]

**Reviewer Response**

**Reviewer #1:**

1. Authors have partially responded my previous comments and without complete corrections there are no further improvisations in the manuscript.

**Explanation:** As per reviewers suggestion we have revised the analysis, result, discussion and conclusion part of the paper.

2. I have major concern in their analysis process. This is a complex survey conducted in India and authors should follow the complex survey analyses on their analyses.

**Explanation:** As per the reviewers valuable suggestion and in view of the complex survey, we have adopted the analysis accordingly. In the multivariate logistic regression analysis we have adjusted the impact of clustering and stratification.

3. I have commented about weights and weights related analyses in the entire manuscript.

**Explanation:** The analysis of the entire data including multivariate regression analysis has been carried out after assigning survey weights that is available in the GATS-1 and GATS-2 datasets. Further details of the weighting procedure are provided in Appendix B on Sample design in the GATS report (9,16).

4. I have noted to provide brief analyses process of entire manuscript which could be published in supplemental file, especially how the numbers were calculated for Rate, Decomposition and Interaction in the tables.

**Explanation:** As per reviewers suggestion we have provided syntax in the supplementary file and added an explanation .

5. Authors did not provide explanations how their models were multivariate. Discussion and conclusion are very hard to follow.

**Explanation:** We have applied Multivariate binary logistic regression model to investigate the adjusted associations of socioeconomic, demographic and knowledge correlates of tobacco consumption in the smoking and smokeless forms in India. In this paper we have applied four multivariate logistic regression models. In all these regression models we have included 18 covariates together to get an adjusted estimates from multivariate model. We have also provided syntax in supplementary file.

As per the suggestion we have revised the discussion and conclusion part.
