# Supplementary material for: Declining trend of smoking and smokeless tobacco in India: A decomposition analysis
Source: PLoS One. 2021 Feb 25;16(2):e0247226. doi: 10.1371/journal.pone.0247226 (PMC7906458; doi:10.1371/journal.pone.0247226)
Supplement: Attachment — Submitted filename: Explanation.docx [file pone.0247226.s004.docx]

**Reviewers Response**

Reviewer #1: How authors applied data weights in their analysis is still not clear. Details are not available in the manuscript. Authors referred reference 9 and 16 Appendix B for weight calculation procedures, but referred document did not provide details about how to use calculated weights. Authors should provide detailed procedures how they applied calculated weights in their entire outcomes. Were they use STRATA, PSU and Weights or STRATA and Weights or PSU and Weights or only Weights? I don’t see any weight related variables in the syntax as well.

Explanation: Survey weight is given in the GATS-1 and 2 data files. Initially we have performed analysis in SPSS software and used syntax weight on. After reviewers suggestion, to adjust complex analysis we have adjusted clustering and stratum effect while doing analysis in STATA-14 software and applied the survey weight to handle the complex survey design.

While generating all the tables of this paper, each record (individual case) was multiplied by survey weight. These weights were estimated for adjustment of 1) unequal probability of selection, 2) differential response rates across states and male/ female in rural/ urban areas within the states and 3) differences in the distribution of survey population and actual population (projected as on survey period) of each state by rural/urban areas and by sex and broad age-group.
In other words, the weights were the adjustment within each individual state and across the states.

Further details of the weighting procedure are provided in section A 4 page 216, on GATS-1 report.

https://www.healis.org/pdf/special-report/GATS_1.pdf

2. I guess, there are different steps in SPSS than other statistical software for complex sample analysis. How they develop .csplan file in SPSS for complex sample or How they prepared “Analysis Preparation Wizard”? Were these all available GATS dataset.

Explanation: Complex analysis adjustment has been done in stata software. We have used the command svyset gatscluster [pweight= gatsweight], strata (gatsstrata). All the mentioned variable are available in GATS dataset.

3. After reading their syntax, I don’t think their logistic regression is multivariate, it should be multivariable logistic regression.

Explanation : The term multivariate and multivariable are often used interchangeably in the public health literature. However, as per the suggestion we have used multivariable logistic regression in manuscript.

4. For decomposition, detailed provided in the supplemental file should be in the Method section instead of that complex equation.

Explanation : As per the journal guideline we have written question in the method section and according to readers ease of understanding question explanation has been given in supplementary file.

5. 4th para of conclusion, limitations of the paper should be incorporated in the Discussion section. There are redundancies in the Conclusions section and its longer than Discussion. Conclusions should be within one short para probably 4 to 5 sentences.

Explanation :As per the suggestion we have revised the conclusion part and added limitation in the discussion part of the paper.
