# Supplementary material for: Declining trend of smoking and smokeless tobacco in India: A decomposition analysis
Source: PLoS One. 2021 Feb 25;16(2):e0247226. doi: 10.1371/journal.pone.0247226 (PMC7906458; doi:10.1371/journal.pone.0247226)
Supplement: Attachment — Submitted filename: Reviewer Response.docx [file pone.0247226.s005.docx]

**Reviewer Response**

**Reviewer #1:** Authors provided following responses to my weight related comments in the response section only but did not included in the manuscript. I would like to suggest authors to include these explanations into the Analysis section of the manuscript, so that readers can understand how authors had done their analysis.

**Explanation:** As per suggestion we have included the explanation in the analysis section of the paper.
